# Diagnostic Efficacy of Carotid Ultrasound for Predicting the Risk of Perioperative Hypotension or Fluid Responsiveness: A Meta-Analysis

**DOI:** 10.3390/diagnostics13132290

**Published:** 2023-07-06

**Authors:** Kuo-Chuan Hung, Yen-Ta Huang, Wen-Wen Tsai, Ping-Heng Tan, Jheng-Yan Wu, Po-Yu Huang, Ting-Hui Liu, I-Wen Chen, Cheuk-Kwan Sun

**Affiliations:** 1 School of Medicine, College of Medicine, National Sun Yat-sen University, Kaohsiung City 80424, Taiwan; ed102605@gmail.com (K.-C.H.); tanphphd@yahoo.com.tw (P.-H.T.); 2 Department of Anesthesiology, Chi Mei Medical Center, Tainan City 71004, Taiwan; 3 Department of Surgery, National Cheng Kung University Hospital, College of Medicine, National Cheng Kung University, Tainan City 70101, Taiwan; 4 Department of Neurology, Chi Mei Medical Center, Tainan City 71004, Taiwan; wenwen102022025@gmail.com; 5 Department of Nutrition, Chi Mei Medical Center, Tainan City 71004, Taiwan; 6 Department of Internal Medicine, Chi Mei Medical Center, Tainan City 71004, Taiwan; 7 Department of General Internal Medicine, Chi Mei Medical Center, Tainan City 71004, Taiwan; 8 Department of Anesthesiology, Chi Mei Medical Center, Liouying, Tainan City 73657, Taiwan; 9 Department of Emergency Medicine, E-Da Dachang Hospital, I-Shou University, Kaohsiung City 82445, Taiwan; 10 School of Medicine for International Students, College of Medicine, I-Shou University, Kaohsiung City 82445, Taiwan

**Keywords:** carotid ultrasound, hypotension, fluid responsiveness, meta-analysis, perioperative

## Abstract

Despite the acceptance of carotid ultrasound for predicting patients’ fluid responsiveness in critical care and anesthesia, its efficacy for predicting hypotension and fluid responsiveness remains unclear in the perioperative setting. Electronic databases were searched from inception to May 2023 to identify observational studies focusing on the use of corrected blood flow time (FTc) and respirophasic variation in carotid artery blood flow peak velocity (ΔVpeak) for assessing the risks of hypotension and fluid responsiveness. Using FTc as a predictive tool (four studies), the analysis yielded a pooled sensitivity of 0.82 (95% confidence interval (CI): 0.72 to 0.89) and specificity of 0.94 (95% CI: 0.88 to 0.97) for the risk of hypotension (area under curve (AUC): 0.95). For fluid responsiveness, the sensitivity and specificity of FTc were 0.79 (95% CI: 0.72 to 0.84) and 0.81 (95% CI: 0.75 to 0.86), respectively (AUC: 0.87). In contrast, the use of ΔVpeak to predict the risk of fluid responsiveness showed a pooled sensitivity of 0.76 (95% CI: 0.63 to 0.85) and specificity of 0.74 (95% CI: 0.66 to 0.8) (AUC: 0.79). The current meta-analysis provides robust evidence supporting the high diagnostic accuracy of FTc in predicting perioperative hypotension and fluid responsiveness, which requires further studies for verification.

## 1. Introduction

Despite the lack of a widely accepted definition, intraoperative hypotension may occur in approximately one-fourth to one-third of patients [1,2]. Its development can be attributed to surgery- (e.g., blood loss and emergency surgery), anesthesia- (e.g., drug-induced vasodilation), or patient-related (e.g., age and cardiovascular disease) factors [3,4,5]. Hypotension-related organ hypoperfusion is known to increase perioperative morbidity and mortality as well as healthcare utilization. Several studies have linked the occurrence of intraoperative hypotension to postoperative nausea/vomiting, myocardial infarction, postoperative delirium, stroke, acute kidney injury, prolonged postoperative hospital stay, and even fatality [3,6,7,8,9,10,11,12,13]. Since the probability of organ damage and other serious complications is known to increase with the duration of hypotension [6], blood pressure monitoring for the prevention of perioperative hypotension is critical for avoiding associated complications. On the other hand, determining fluid responsiveness, which refers to the ability of the cardiovascular system to respond to fluid administration by boosting cardiac output, is also crucial for optimizing fluid balance and improving hemodynamic stability in clinical practice [14,15]. In clinical practice, methods such as inferior vena cava (IVC) assessment [16,17], passive leg raising [18], pulse pressure variation (PPV), and stroke volume variation (SVV) are commonly used to assess fluid responsiveness. While IVC assessment evaluates IVC diameter and collapsibility to determine intravascular volume status [16,17], passive leg raising temporarily increases venous return to observe changes in cardiac output [18]. On the other hand, PPV and SVV, which are predictors of fluid responsiveness derived from arterial waveform analysis, reflect respiratory cycle-related variations in pulse pressure and stroke volume, respectively. An accurate fluid status assessment enables clinicians to adopt an individualized strategy to achieve a delicate balance between adequate tissue perfusion and fluid overload, which is especially important in critically ill individuals with hemodynamic instability.

In recent years, the corrected blood flow time (FTc) measured in the carotid artery has emerged as a potential predictor of fluid responsiveness in both spontaneously breathing and mechanically ventilated patients [19,20,21]. Although a recent meta-analysis focusing on patients in critical and anesthesia settings has provided further support regarding the diagnostic efficacy of FTc for predicting fluid responsiveness [22], its enrollment of a mixed population of study subjects including those with septic shock could not exclusively reflect the role of FTc in anesthetized patients. Furthermore, besides being an indicator of fluid responsiveness, a number of studies have suggested the usefulness of FTc as a predictor of perioperative hypotension [23,24]. Nevertheless, the perioperative applicability of FTc for predicting the risk of hypotension and fluid responsiveness remained questionable because of a lack of systematic evaluation. Therefore, the aim of the present meta-analysis is to address this issue through systematically reviewing currently available observational studies focusing on the use of FTc in the perioperative setting.

## 2. Materials and Methods

### 2.1. Study Protocol

This systematic review, which followed the Preferred Reporting Items for Systematic Reviews and Meta-Analyses of Diagnostic Test Accuracy Studies (PRISMA-DTA) guideline, was registered at PROSPERO (CRD42023425534).

### 2.2. Data Source and Literature Search

The present study involved two independent authors who conducted a comprehensive search in four databases, namely, Medline, Cochrane Library, Embase, and Google Scholar, from their inception until 12 May 2023 to identify relevant studies utilizing Doppler ultrasound-derived parameters, including FTc and respirophasic variation in carotid artery blood flow peak velocity (ΔVpeak) for predicting the likelihood of hypotension and fluid responsiveness in the perioperative setting. No restrictions were imposed on language and country of publication. Google Scholar was used as an additional resource alongside established academic databases to maximize the comprehensiveness of our search to provide a more extensive overview of the available evidence. For our literature search, the following keywords were utilized: (“General anesthesia” or “Surgery” or “Surgical procedures” or “Regional anesthesia” or “Spinal anesthesia” or “Epidural anesthesia”) and (“Carotid artery” or “carotid artery-corrected flow time” or “respiratory variations of peak blood flow velocity” or “respirophasic variation in blood flow peak velocity”) and (“Sonography” or “Echography” or “Ultrasonographic” or “Ultrasonography” or “Ultrasound”) and (“Hypotension” or “Fluid responsiveness” or “Fluid challenge” or “Dehydration” or “hypotensive”). In addition to the database search, manual screening was conducted to retrieve potentially eligible studies. All disagreements or conflicts between the two authors were resolved through a consensus that involved a third investigator. Further details on the search strategy employed for one of the databases (i.e., Medline) can be found in Supplemental Table S1.

### 2.3. Inclusion and Exclusion Criteria

The inclusion of studies was determined based on the following criteria: (a) adult patients in whom carotid ultrasound measurements were performed in the perioperative setting regardless of the anesthetic techniques; (b) use of carotid artery-derived sonographic parameters for predicting hypotension or fluid responsiveness; (c) and availability of details pertaining to sensitivity, specificity, number of patients with hypotension or fluid responsiveness. For the current meta-analysis, randomized controlled trials, cohort studies, and case-control studies were all considered eligible.

Studies that met any of the following criteria were excluded: (a) reported solely as case series, abstracts, case reports, conference papers, or review articles; (b) focused on non-surgical patients or pediatric populations; (c) lacked outcomes of interest; (d) or unavailability of a full-text version.

### 2.4. Data Extraction

Two authors independently extracted data from individual studies with all disagreements resolved through the involvement of a third investigator. The following data were collected: name of the first author, study characteristics (e.g., sample size, setting), patient demographics (including age and gender), sensitivity and specificity, carotid artery-related parameters (i.e., FTc and ΔVpeak), number of patients with hypotension or fluid responsiveness, and country of origin. Efforts were made to obtain missing information by contacting the authors of those articles.

### 2.5. Outcomes and Definitions

The primary objective of this meta-analysis was to evaluate the diagnostic accuracy of FTc in predicting the likelihood of perioperative hypotension, with secondary outcomes being focused on the diagnostic efficacy of FTc or ΔVpeak for predicting perioperative fluid responsiveness. FTc represents the temporal interval of left ventricular ejection measured between the initiation of upstroke and the incisural notch on a systolic pulse waveform [25]. On the other hand, ΔVpeak signifies the alteration in the amplitude of the carotid pulse upstroke in response to respiratory fluctuations. The diagnostic criteria for hypotension and fluid responsiveness were in accordance with those employed in individual studies. Events of perioperative hypotension or fluid responsiveness were defined as those that occurred during the preoperative, intraoperative, or postoperative phase. For studies that compared FTc or ΔVpeak in patients receiving mechanical ventilation with different tidal volumes (e.g., 6 mL/kg to 10 mL/kg), we adopted the data acquired at or close to a tidal volume of 10 mL/kg to simulate the real-world clinical scenario.

### 2.6. Quality Assessment

The Quality Assessment for Diagnostic Accuracy Studies-2 (QUADAS-2) tool, which comprises two categories, “risk of bias” and “applicability concerns”, was employed to evaluate the quality of each included study [26]. The former category consists of four domains, while the latter contains three domains. Both authors conducted a subjective review of all included studies and assigned ratings of “low risk,” “some concerns,” or “high risk” to each domain. All discrepancies were addressed through discussion until a consensus was reached. A third author was involved if necessary.

### 2.7. Statistical Analysis

In the present study, diagnostic performance was assessed by calculating the area under the curve (AUC) from a summary receiver operating characteristic (sROC) curve [27], which is a widely used measure of overall accuracy in diagnostic tests. In addition, the post-test probability was examined with Fagan’s nomogram, which is a graphical tool that integrates the pre-test probability of a disease with the likelihood ratios (LRs) of the test results to estimate the post-test probability. To evaluate the potential publication bias, Deek’s funnel plot was examined, which is a graphical method that assesses the relationship between the standard error and effect size of each study [28]. A significance level of 0.05 was employed to determine statistical significance. All statistical analyses were conducted using the MIDAS command in Stata 15 (StataCorp LLC., College Station, TX, USA).

## 3. Results

### 3.1. Study Selection and Study Characteristics

**Figure 1:**
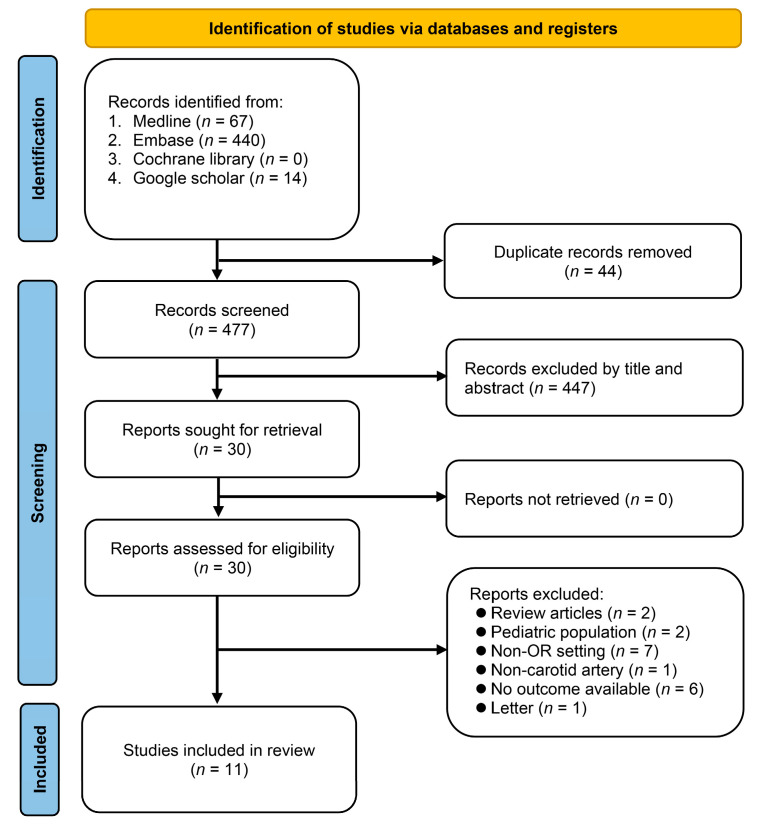
PRISMA flow diagram. OR: operating room.

A comprehensive systematic search of four electronic databases, namely, Medline, Embase, Cochrane Library, and Google Scholar, resulted in the retrieval of 521 records. After removing duplicate records (*n* = 44) and those deemed unsuitable after screening their titles and abstracts (*n* = 477), a total of 30 reports were identified as potentially eligible for further analysis. Further exclusion of review articles, studies involving pediatric populations, those conducted in non-operating room settings, non-carotid artery studies, and those lacking available outcome data gave 11 studies for final review (Figure 1) [19,20,21,23,24,29,30,31,32,33,34].

Table 1 presents a summary of patient characteristics in the 11 studies, all of which adopted a prospective observational design. The mean or median age of the patients varied from 35 to 79.1 years. Eight of the studies involved a mixed-gender population with a male prevalence ranging from 34% to 67.5% [19,20,21,23,24,29,31,33]. Interestingly, two studies exclusively focused on female patients undergoing cesarean section [30,34], while one study did not provide relevant information regarding gender distribution [32]. The sample sizes across the studies ranged from 35 to 112 participants. Regarding the timing of carotid ultrasonic measurements, the assessments were conducted before anesthesia in four studies [19,21,23,24], after anesthetic induction in three studies [31,32,33], during the postoperative period in one study [20], and before regional anesthesia in three studies [29,30,34]. The incidence of hypotension or fluid responsiveness also varied among the studies, ranging from 42.4% to 63.6%. The cut-off values for FTc ranged from 313.8 to 379.1 (323.4 to 379.1 for hypotension; 313.8 to 356.5 for fluid responsiveness), while those for ΔVpeak varied from 7.5% to 11.69%. The studies included in the present analysis were conducted in three countries, namely, China (*n* = 6) [19,24,29,32,33,34], Korea (*n* = 4) [20,21,30,31], and India (*n* = 1) [23], indicating an Asian predominance.

**Figure 2:**
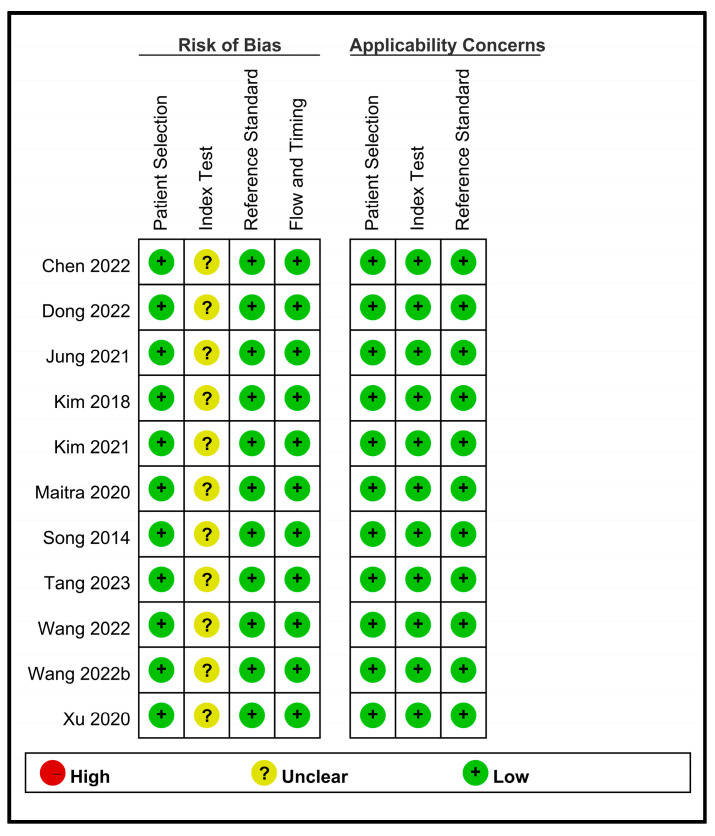
An overview of the risk of bias and applicability concerns of the 11 included studies [19,20,21,23,24,29,30,31,32,33,34].

Figure 2 provides a comprehensive overview of the risk of bias and applicability concerns of the 11 included studies. Regarding the four domains pertaining to the risk of bias, all studies were regarded as having a low risk of bias in the domains of patient selection, reference standard, as well as flow and timing. However, all studies were considered to have some concerns in relation to the domain of the index test. Focusing on applicability concerns, all studies were regarded as having a low risk in the three domains of patient selection, index test, and reference standard.

### 3.2. Outcomes

#### 3.2.1. Diagnostic Efficacy of Carotid Ultrasonography for Predicting Perioperative Hypotension and Fluid Responsiveness

**Figure 3:**
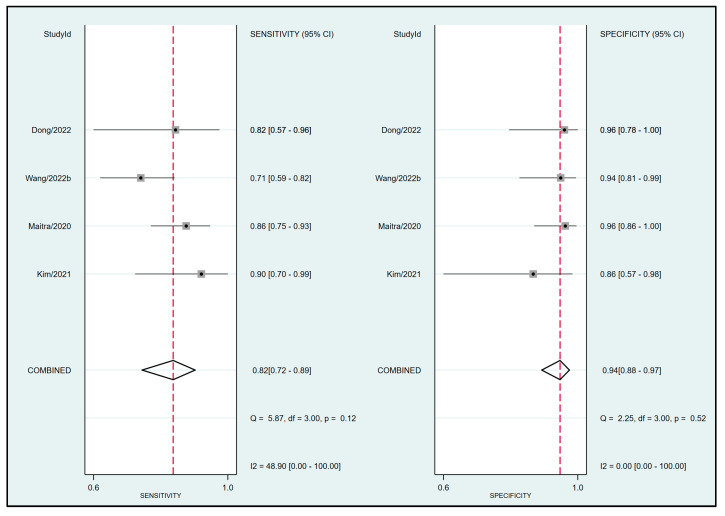
Forest plot showing the pooled sensitivity and specificity of corrected blood flow time (FTc) measured in the carotid artery for predicting perioperative hypotension [23,24,29,30].

**Figure 4:**
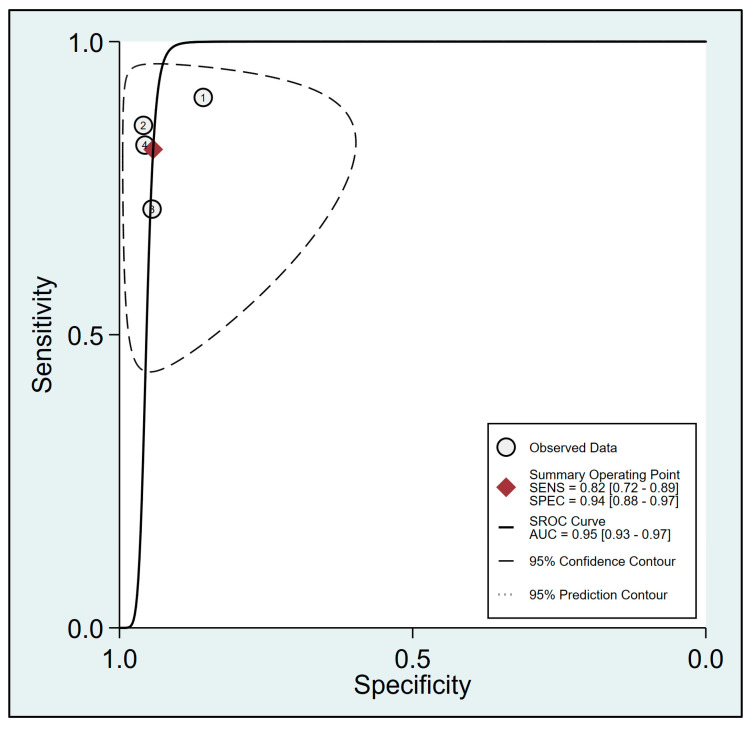
Summary receiver operating characteristic (sROC) curve analysis of sensitivity and specificity of corrected blood flow time (FTc) for predicting perioperative hypotension [23,24,29,30]. The solid line denotes weighted sROC, while the open circles represent estimates of sensitivity and 1−specificity of different studies with pooled point estimates of outcomes shown as diamonds. AUC: area under the curve; SENS: sensitivity; SPEC: specificity.

To assess the effectiveness of FTc for predicting the risk of perioperative hypotension, a meta-analysis was conducted using data from four relevant studies [23,24,29,30]. The analysis yielded a pooled sensitivity of 0.82 (95% CI: 0.72 to 0.89, I^2^ = 48.9%) and a pooled specificity of 0.94 (95% CI: 0.88 to 0.97, I^2^ = 0%) for FTc as a predictive tool, as demonstrated in Figure 3. Furthermore, the pooled AUC was 0.95 (95% CI: 0.93 to 0.97) (Figure 4).

**Figure 5:**
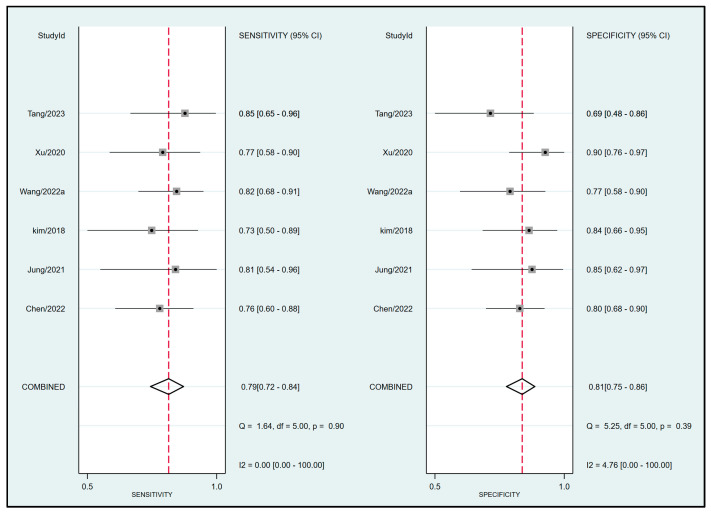
Forest plot depicting the pooled sensitivity and specificity of corrected blood flow time (FTc) measured in the carotid artery for predicting perioperative fluid responsiveness [19,20,21,32,33,34].

**Figure 6:**
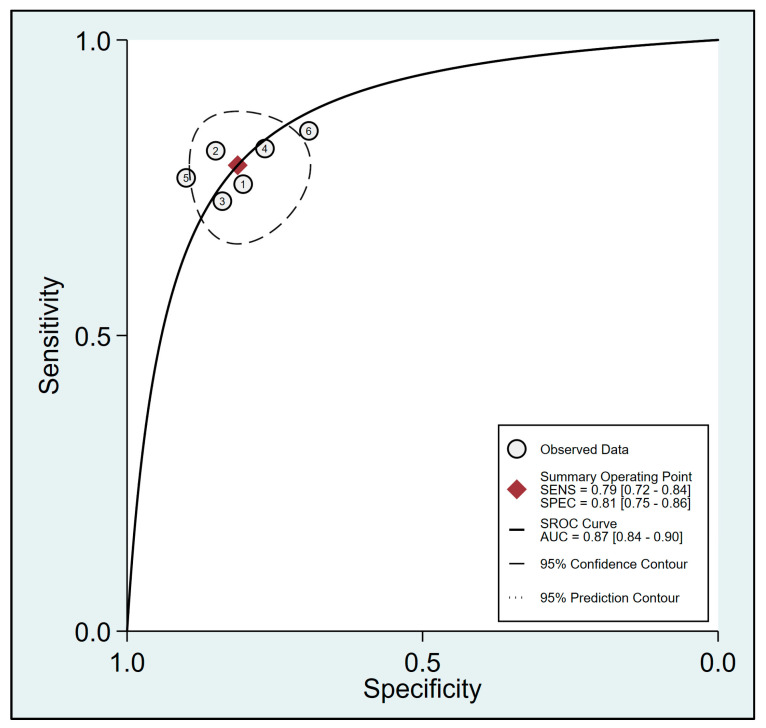
Summary receiver operating characteristic (sROC) curve analysis of sensitivity and specificity of corrected blood flow time (FTc) for predicting perioperative fluid responsiveness [19,20,21,32,33,34]. The solid line represents weighted sROC, while the open circles denote estimates of sensitivity and 1−specificity of different studies with pooled point estimates of outcomes expressed as diamonds. AUC: area under the curve; SENS: sensitivity; SPEC: specificity.

**Figure 7:**
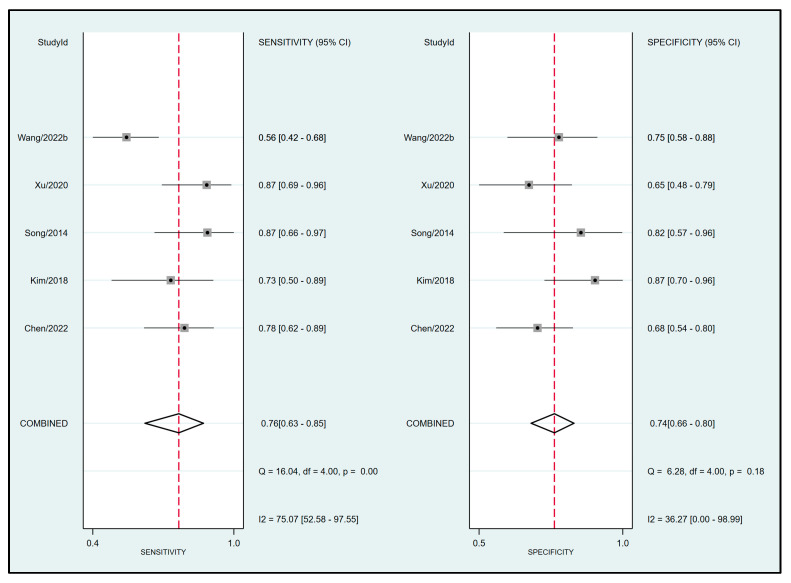
Forest plot demonstrating the pooled sensitivity and specificity of respirophasic variation in carotid artery blood flow peak velocity (ΔVpeak) for predicting perioperative fluid responsiveness [19,21,24,31,34].

**Figure 8:**
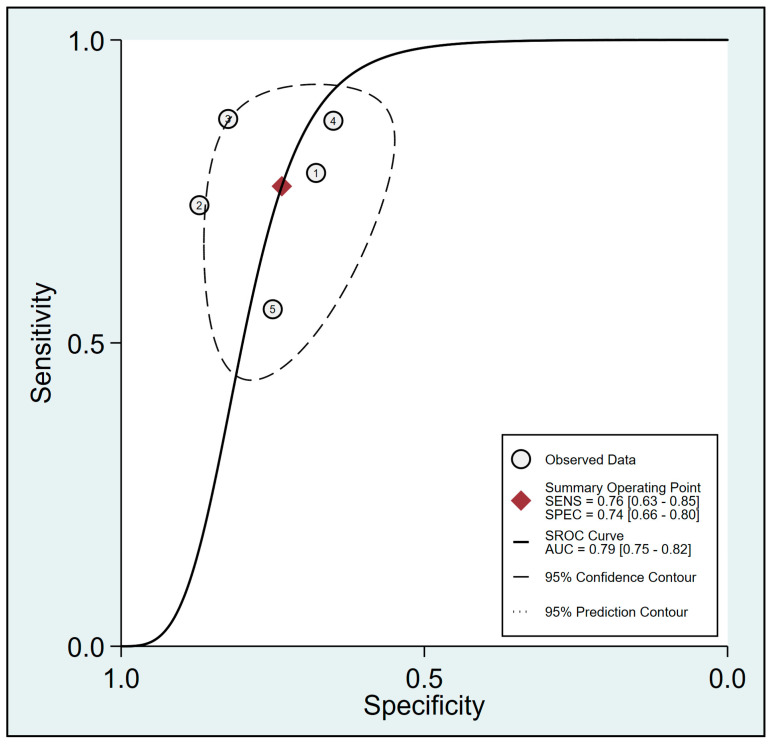
Summary receiver operating characteristic (sROC) curve analysis of sensitivity and specificity of respirophasic variation in carotid artery blood flow peak velocity (ΔVpeak) for predicting perioperative fluid responsiveness [19,21,24,31,34]. The solid line denotes weighted sROC, while the open circles represent estimates of sensitivity and 1−specificity of different studies with pooled point estimates of outcomes shown as diamonds. AUC: area under the curve; SENS: sensitivity; SPEC: specificity.

Pooled results from six studies [19,20,21,32,33,34] examining the relationship between FTc and perioperative fluid responsiveness showed an estimated sensitivity and specificity of 0.79 (95% CI: 0.72 to 0.84, I^2^ = 0%) and 0.81 (95% CI: 0.75 to 0.86, I^2^ = 4.76%), respectively, for FTc as a predictive tool (Figure 5) [19,20,21,32,33,34]. The pooled AUC was 0.87 (95% CI: 0.84 to 0.9) (Figure 6). In contrast, the use of ΔVpeak to predict the risk of fluid responsiveness demonstrated a pooled sensitivity of 0.76 (95% CI: 0.63 to 0.85, I^2^ = 75.07%) and a pooled specificity of 0.74 (95% CI: 0.66 to 0.8, I^2^ = 36.27%) (Figure 7) [19,21,24,31,34]. Regarding diagnostic performance, the AUC was 0.79 (95% CI: 0.75 to 0.82) (Figure 8).

#### 3.2.2. Publication Bias

The results of Deek’s funnel plot asymmetry test indicated a low risk of publication bias for the association of FTc with perioperative hypotension or fluid responsiveness (*p* = 0.81 and *p* = 0.56, respectively) (Supplemental Figures S1 and S2). However, a potential risk of bias was detected in the correlation between ΔVpeak and perioperative fluid responsiveness (Supplemental Figure S3).

#### 3.2.3. Fagan Nomogram for Post-Test Probabilities

**Figure 9:**
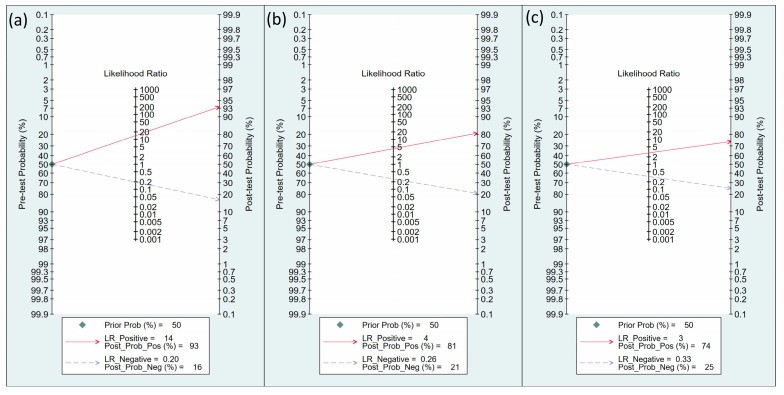
Fagan’s nomogram for assessing the clinical utility of corrected blood flow time (FTc) in predicting perioperative (**a**) hypotension and (**b**) fluid responsiveness, as well as (**c**) respirophasic variation in carotid artery blood flow peak velocity (ΔVpeak) in predicting perioperative fluid responsiveness.

The efficacy of FTc as a predictor of perioperative hypotension and fluid responsiveness was evaluated using Fagan nomograms. Focusing on perioperative hypotension, the positive and negative likelihood ratios were 14 and 0.2, respectively. Assuming a pre-test probability of 50%, the introduction of FTc as a diagnostic test resulted in post-test probabilities of 93% for a positive result and 16% for a negative result (Figure 9a). Similarly, for the prediction of perioperative fluid responsiveness, the positive likelihood and negative likelihood ratios of FTc were 4 and 0.26, respectively, based on Fagan nomograms. Assuming a pre-test probability of 50%, the post-test probabilities of the event were 81% for a positive result and 21% for a negative result (Figure 9b). On the other hand, the use of ΔVpeak as a predictor of perioperative fluid responsiveness showed a positive likelihood ratio of 3 and a negative likelihood ratio of 0.33. Assuming a pre-test probability of 50%, the use of Δvpeak as a diagnostic test yielded post-test probabilities of 74% for a positive result and 25% for a negative result (Figure 9c).

## 4. Discussion

This meta-analysis evaluated the applicability of carotid ultrasound in predicting the risks of hypotension and dehydration during the perioperative period through an analysis of 11 studies involving patients aged 35–79.1 years. The FTc exhibited a pooled sensitivity of 0.82 and specificity of 0.94, indicating a high diagnostic accuracy with an AUC of 0.95 based on an sROC curve. Furthermore, an examination of the predictive capacity of FTc for perioperative fluid responsiveness from a subset of six studies demonstrated an estimated sensitivity of 0.79 and specificity of 0.81, as well as a pooled AUC of 0.87. Conversely, the pooled sensitivity and specificity of ΔVpeak as a predictor of fluid responsiveness were 0.76 and 0.74, respectively, with an AUC of 0.79.

The impact of intraoperative hypotension on patient outcomes has previously been addressed in the current literature. A recent study of 605 elderly patients undergoing thoracic and orthopedic surgeries showed a correlation between intraoperative hypotension (mean arterial blood pressure (MAP) ≤ 65 mmHg) lasting for five minutes or more and an elevated incidence of postoperative delirium [6]. Consistently, utilizing data from a US electronic health record database, a retrospective multicenter cohort study comprising 112,912 noncardiac and non-obstetric surgeries found a significant association between intraoperative hypotension at MAP ≤ 55 mmHg and persistent acute kidney dysfunction [10]. In addition, a previous investigation demonstrated a link between postoperative myocardial injury and intraoperative hypotension, defined as a decrease of at least 40% from the pre-induction MAP with a cumulative duration exceeding 30 min, in elderly patients receiving vascular surgery [12]. Such findings have underscored the importance of accurate prediction and prevention of intraoperative hypotension.

In the current meta-analysis, the use of FTc for hypotension prediction exhibited a pooled sensitivity of 0.82 and specificity of 0.94, suggesting a high diagnostic accuracy. In addition to carotid ultrasound, recent studies have highlighted the value of the Hypotension Prediction Index, which is an invasive approach utilizing arterial waveform features with a sensitivity and specificity of 81.7% and 81.7%, respectively, for predicting hypotension 10 min before the occurrence of events [35]. Two recent randomized controlled trials found that a significant reduction in intraoperative hypotension compared to standard care could be achieved through incorporating the Hypotension Prediction Index and other hemodynamic variables into a machine learning-based early warning system (EWS) with a hemodynamic algorithm [36,37]. Additionally, this intervention was associated with lower levels of biomarkers related to organ injury and oxidative stress [36]. These results highlight the clinical significance of utilizing such a monitoring tool (e.g., Hypotension Prediction Index) to prevent intraoperative hypotension and its associated complications. Despite similar diagnostic capabilities between FTc and Hypotension Prediction Index, carotid ultrasound possesses the added benefits of simultaneous risk assessment for both hypotension and fluid responsiveness as well as non-invasiveness. A previous meta-analysis conducted in anesthesia and critical care settings has reported comparable pooled sensitivity and specificity between FTc and ΔVpeak in predicting fluid responsiveness [22]. However, our findings indicated that FTc exhibited a superior diagnostic efficacy compared to ΔVpeak specifically in the anesthesia setting. The observed discrepancy could be attributed to variations in the studied population such as the inclusion of patients with critical illnesses (e.g., septic shock) that may impact the diagnostic performance of ΔVpeak for fluid responsiveness in the previous meta-analysis [22].

Although PPV and SVV are conventional hemodynamic parameters widely used to evaluate fluid responsiveness in patients [38,39,40], their utilizations require invasive arterial catheterization, which may not be feasible in all clinical scenarios. In addition, despite the accuracy of these two methods in predicting fluid responsiveness in mechanically ventilated patients who receive a controlled tidal volume ventilation [41,42], their reliability may be compromised in patients who are not mechanically ventilated or those with spontaneous breathing. Moreover, the presence of arrhythmias or irregular cardiac cycles can significantly influence the precision of these parameters, thereby diminishing their reliability as perioperative hemodynamic indicators [43].

In contrast, the utilizations of carotid ultrasound-derived FTc and ΔVpeak offer several notable advantages. First, this technique is completely non-invasive and can be easily assessed using carotid ultrasound. Second, our findings suggested the potential usefulness of pre-anesthesia real-time ultrasound examination in the prevention of hypotension following induction, thereby enhancing patient safety and avoiding invasive procedures (e.g., arterial catheterization). Third, these parameters are applicable even in the setting of a low tidal volume (e.g., 6 mL/kg) or in patients with spontaneous breathing, unlike other dynamic markers (e.g., PPV) that mandate mechanical ventilation with a tidal volume of no less than 8 mL/kg for the precise prediction of fluid responsiveness [44,45]. In addition, intrathoracic pressure variations during respiration have no significant impact on carotid FTc measurements [46]. Nevertheless, despite the potential advantages of FTc and ΔVpeak, continuous real-time prediction of the risk of future hypotension or fluid responsiveness using carotid ultrasonography may not be clinically feasible. Furthermore, an accurate prediction of hemodynamic status with parameters such as FTc and ΔVpeak can be challenging to novice sonologists [47,48], thereby potentially hindering their clinical applicability.

Multiple risk factors have been recognized as contributors to intraoperative hypotension, including an older age (≥60 years), elevated ASA physical status, emergency surgical procedures, and increased body mass index, as well as hypertension history and an estimated blood loss surpassing 500 mL [2,49]. Given the high diagnostic effectiveness of carotid ultrasound based on the current meta-analysis, incorporating such identified risk factors into the diagnostic framework may further improve its predictive accuracy for intraoperative hypotension. Future research may focus on exploring this issue.

The current investigation had several noteworthy limitations. First, variations in anesthetic techniques and timing of ultrasonic measurements across the included studies may introduce potential bias that could potentially affect the efficacy of carotid ultrasound as a predictive tool. Second, provided that perioperative hypotension is more likely in older patients and those with comorbidities, the inclusion of those with a mean or median age below 70 in nine studies as well as the predominant recruitment of relatively healthy participants (i.e., ASA Class I or II) in the included studies may obscure the significance of our findings. Third, the relatively small sample sizes ranging from 35 to 112 in the included reports may limit the statistical power and precision of our analysis. Fourth, the inclusion of only Asian populations (i.e., China, Korea, and India) may restrict the extrapolation of our findings to individuals of different ethnic backgrounds and countries of limited equipment availability. Finally, a recent study has reported the measurement of respiratory variation in internal jugular vein diameter using a portable ultrasound as a non-invasive bedside tool for volume status evaluation, highlighting the objectivity of information about fluid status, its ease of use, and a short learning curve [50]. However, the lack of direct comparative data between such an approach and the measurement of FTc warrants further evaluation and investigation to compare their individual strengths and limitations in fluid status assessment.

## 5. Conclusions

The current meta-analysis not only provides robust evidence supporting the high diagnostic accuracy of FTc in predicting perioperative hypotension as demonstrated by its pooled sensitivity of 0.82, specificity of 0.94, and AUC of 0.95, but also identifies FTc as a promising predictor of perioperative fluid responsiveness with a sensitivity of 0.79, specificity of 0.81, and pooled AUC of 0.87. In contrast, ΔVpeak exhibited relatively low sensitivity (0.76), specificity (0.74), and AUC (0.79) as a fluid responsiveness predictor. Future studies are warranted to investigate the correlation between FTc and clinical outcomes such as postoperative complications or length of hospital stay, as well as further explore the effectiveness of combining FTc with other predictive tools to enhance the accuracy of perioperative risk assessment.

## Figures and Tables

**Table 1:** Characteristics of studies (*n* = 11).

Studies	Age (Years) †	Male (%)	*N*	ASA-PS	Type of Study	Time of Ultrasonic Measurement	Outcome (Incidence%)	Cut-Off Value FTc (ms)	Country
Chen 2022 [19]	71.6	62.9	97	16/52/29	Pro	Before AI	FR (42.4)	340.74	China
Dong 2022 [29]	79.1 vs. 71.2 ‡	52.5	40	0/31/9	Pro	Before SA	Hypo (43)	323.4	China
Jung 2021 [20]	55.0	61.1	36	13/18/5	Pro	Post-op	FR (44.4)	345.1	Korea
Kim 2018 [21]	54.0	34	53	28/21/4	Pro	Before AI	FR (42)	349.4	Korea
Kim 2021 [30]	35	0	35	NA ⁋	Pro	Before SA	Hypo (60)	346.4	Korea
Maitra 2020 [23]	40.7	48.2	112	63/47/0	Pro	Before AI	Hypo (56.3)	330.2	India
Song 2014 [31]	66 vs. 67 ‡	67.5	40	NA	Pro	After AI	FR (57.50)	-	Korea
Tang 2023 [32]	50.98	NA	52	0/43/9	Pro	After AI	FR (50)	356.5	China
Wang 2022a [33]	29.3 vs 28.2 ‡	49.4	79	0/40/29	Pro	After AI	FR (62)	331.93	China
Wang 2022b [24]	69 vs. 68 ‡	65.7	99	88/11/0	Pro	Before AI	Hypo (63.6)	379.1	China
Xu 2020 [34]	33 vs. 34 ‡	0	70	50/20/0	Pro	Before SA and EA	FR (42.90)	313.8	China

FR: fluid responsiveness; † mean or median; ‡ data presented as those with hypotension or fluid responsiveness vs. those without; AI: anesthesia induction; Post-op: postoperative period; ⁋ only the inclusion of patients with ASA I or II; *N*: total number of patients; Hypo: hypotension; FTc: corrected flow time; ASA-PS: American Society of Anesthesiologists physical status; Pro: prospective observational study.

## Data Availability

The original contributions presented in this study are included in this article/Supplementary Material, further inquiries can be directed to the corresponding authors.

## Supplementary Materials

The following supporting information can be downloaded at https://www.mdpi.com/article/10.3390/diagnostics13132290/s1, Figure S1: Deek’s funnel plot asymmetry test indicated a low risk of publication bias for the association of corrected blood flow time (FTc) with perioperative hypotension; Figure S2: Deek’s funnel plot asymmetry test indicated a low risk of publication bias for the association of corrected blood flow time (FTc) with perioperative fluid responsiveness; Figure S3: Deek’s funnel plot asymmetry test indicated a potential risk of publication bias for the association of respirophasic variation in carotid artery blood flow peak velocity (ΔVpeak) with perioperative fluid responsiveness; Table S1: Search strategies for Medline.

## References

[1] Czajka S., Putowski Z., Krzych Ł.J. (2023). Post-induction hypotension and intraoperative hypotension as potential separate risk factors for the adverse outcome: A cohort study. J. Anesth..

[2] Temesgen N., Fenta E., Eshetie C., Gelaw M. (2021). Early intraoperative hypotension and its associated factors among surgical patients undergoing surgery under general anesthesia: An observational study. Ann. Med. Surg..

[3] Huang X., Lu X., Guo C., Lin S., Zhang Y., Zhang X., Cheng E., Liu J. (2023). Effect of preoperative risk on the association between intraoperative hypotension and postoperative acute kidney injury in cardiac surgery. Anaesth. Crit. Care Pain Med..

[4] Bijker J.B., van Klei W.A., Kappen T.H., van Wolfswinkel L., Moons K.G., Kalkman C.J. (2007). Incidence of intraoperative hypotension as a function of the chosen definition: Literature definitions applied to a retrospective cohort using automated data collection. Anesthesiology.

[5] Schonberger R.B., Dai F., Michel G., Vaughn M.T., Burg M.M., Mathis M., Kheterpal S., Akhtar S., Shah N., Bardia A. (2022). Association of propofol induction dose and severe pre-incision hypotension among surgical patients over age 65. J. Clin. Anesth..

[6] Duan W., Zhou C.M., Yang J.J., Zhang Y., Li Z.P., Ma D.Q., Yang J.J. (2023). A long duration of intraoperative hypotension is associated with postoperative delirium occurrence following thoracic and orthopedic surgery in elderly. J. Clin. Anesth..

[7] Putowski Z., Majewska K., Gruca K., Zimnoch A., Szczepańska A., Krzych Ł.J., Jabłońska B., Mrowiec S. (2023). Intraoperative Hypotension and Its Association with Postoperative Acute Kidney Injury in Patients Undergoing Pancreaticoduodenectomy: A 5-Year, Single-Center, Retrospective Cohort Study. Med. Sci. Monit. Int. Med. J. Exp. Clin. Res..

[8] Maleczek M., Laxar D., Geroldinger A., Kimberger O. (2023). Intraoperative Hypotension Is Associated with Postoperative Nausea and Vomiting in the PACU: A Retrospective Database Analysis. J. Clin. Med..

[9] Zhang Y., Li S., Li Z., Chen J., Tan H. (2022). Intraoperative Diastolic Hypotension-Prolonged Postoperative Hospital Stay in Patients with Gastric Cancer: A Retrospective Cohort Study with Propensity Score Matching. Int. J. Gen. Med..

[10] Shaw A.D., Khanna A.K., Smischney N.J., Shenoy A.V., Boero I.J., Bershad M., Hwang S., Chen Q., Stapelfeldt W.H. (2022). Intraoperative hypotension is associated with persistent acute kidney disease after noncardiac surgery: A multicentre cohort study. Br. J. Anaesth..

[11] Bijker J.B., van Klei W.A., Vergouwe Y., Eleveld D.J., van Wolfswinkel L., Moons K.G., Kalkman C.J. (2009). Intraoperative hypotension and 1-year mortality after noncardiac surgery. Anesthesiology.

[12] van Waes J.A., van Klei W.A., Wijeysundera D.N., van Wolfswinkel L., Lindsay T.F., Beattie W.S. (2016). Association between Intraoperative Hypotension and Myocardial Injury after Vascular Surgery. Anesthesiology.

[13] Sun L.Y., Chung A.M., Farkouh M.E., van Diepen S., Weinberger J., Bourke M., Ruel M. (2018). Defining an Intraoperative Hypotension Threshold in Association with Stroke in Cardiac Surgery. Anesthesiology.

[14] Nugent K., Berdine G., Pena C. (2022). Does Fluid Administration Based on Fluid Responsiveness Tests such as Passive Leg Raising Improve Outcomes in Sepsis?. Curr. Cardiol. Rev..

[15] Monnet X., Marik P.E., Teboul J.L. (2016). Prediction of fluid responsiveness: An update. Ann. Intensive Care.

[16] La Via L., Astuto M., Dezio V., Muscarà L., Palella S., Zawadka M., Vignon P., Sanfilippo F. (2022). Agreement between subcostal and transhepatic longitudinal imaging of the inferior vena cava for the evaluation of fluid responsiveness: A systematic review. J. Crit. Care.

[17] Orso D., Paoli I., Piani T., Cilenti F.L., Cristiani L., Guglielmo N. (2020). Accuracy of Ultrasonographic Measurements of Inferior Vena Cava to Determine Fluid Responsiveness: A Systematic Review and Meta-Analysis. J. Intensive Care Med..

[18] Monnet X., Marik P., Teboul J.L. (2016). Passive leg raising for predicting fluid responsiveness: A systematic review and meta-analysis. Intensive Care Med..

[19] Chen Y., Liu Z., Fang J., Xie Y., Zhang M., Yang J. (2022). Correlation of carotid corrected flow time and respirophasic variation in blood flow peak velocity with stroke volume variation in elderly patients under general anaesthesia. BMC Anesthesiol..

[20] Jung S., Kim J., Na S., Nam W.S., Kim D.H. (2021). Ability of Carotid Corrected Flow Time to Predict Fluid Responsiveness in Patients Mechanically Ventilated Using Low Tidal Volume after Surgery. J. Clin. Med..

[21] Kim D.H., Shin S., Kim N., Choi T., Choi S.H., Choi Y.S. (2018). Carotid ultrasound measurements for assessing fluid responsiveness in spontaneously breathing patients: Corrected flow time and respirophasic variation in blood flow peak velocity. Br. J. Anaesth..

[22] Singla D., Gupta B., Varshney P., Mangla M., Walikar B.N., Jamir T. (2022). Role of carotid corrected flow time and peak velocity variation in predicting fluid responsiveness: A systematic review and meta-analysis. Korean J. Anesthesiol..

[23] Maitra S., Baidya D.K., Anand R.K., Subramanium R., Bhattacharjee S. (2020). Carotid Artery Corrected Flow Time and Respiratory Variations of Peak Blood Flow Velocity for Prediction of Hypotension After Induction of General Anesthesia in Adult Patients Undergoing Elective Surgery: A Prospective Observational Study. J. Ultrasound Med. Off. J. Am. Inst. Ultrasound Med..

[24] Wang J., Li Y., Su H., Zhao J., Tu F. (2022). Carotid artery corrected flow time and respiratory variations of peak blood flow velocity for prediction of hypotension after induction of general anesthesia in elderly patients. BMC Geriatr..

[25] Hassan S., Turner P. (1983). Systolic time intervals: A review of the method in the non-invasive investigation of cardiac function in health, disease and clinical pharmacology. Postgrad. Med. J..

[26] Whiting P.F., Rutjes A.W., Westwood M.E., Mallett S., Deeks J.J., Reitsma J.B., Leeflang M.M.G., Sterne J.A.C., Bossuyt P.M.M., the QUADAS-2 Group (2011). QUADAS-2: A revised tool for the quality assessment of diagnostic accuracy studies. Ann. Intern. Med..

[27] Jones C.M., Athanasiou T. (2005). Summary receiver operating characteristic curve analysis techniques in the evaluation of diagnostic tests. Ann. Thorac. Surg..

[28] Tsai W.-W., Hung K.-C., Huang Y.-T., Yu C.-H., Lin C.-H., Chen I.-W., Sun C.-K. (2023). Diagnostic efficacy of sonographic measurement of laryngeal air column width difference for predicting the risk of post-extubation stridor: A meta-analysis of observational studies. Front. Med..

[29] Dong H., Geng Z., Zhang Y., Liu W., Lu X., Yang Y. (2022). Predictive value of carotid artery corrected blood flow time and heart rate in elderly patients with hypotension after subarachnoid block. J. Clin. Anesthesiol..

[30] Kim H.J., Choi Y.S., Kim S.H., Lee W., Kwon J.Y., Kim D.H. (2021). Predictability of preoperative carotid artery-corrected flow time for hypotension after spinal anaesthesia in patients undergoing caesarean section: A prospective observational study. Eur. J. Anaesthesiol..

[31] Song Y., Kwak Y.L., Song J.W., Kim Y.J., Shim J.K. (2014). Respirophasic carotid artery peak velocity variation as a predictor of fluid responsiveness in mechanically ventilated patients with coronary artery disease. Br. J. Anaesth..

[32] Tang X., Chen Q., Huang Z., An R., Liang J., Liu H. (2023). Comparison of the carotid corrected flow time and tidal volume challenge for assessing fluid responsiveness in robot-assisted laparoscopic surgery. medRxiv.

[33] Wang H., Chen W., Cheng H., Liu C., Yao W., Ding F., Wang Y., Chen Y. (2022). Value of corrected flow time in common carotid artery in predicting volume responsiveness under mechanical ventilation. Shock.

[34] Xu L., Dai S., Shen J., Lv C., Tang Y., Chen X. (2020). The predictive ability of carotid artery corrected flow time and respirophasic variation in blood flow peak velocity measured by ultrasonography for fluid responsiveness in parturients for cesarean delivery. Minerva Anestesiol..

[35] Davies S.J., Vistisen S.T., Jian Z., Hatib F., Scheeren T.W.L. (2020). Ability of an Arterial Waveform Analysis-Derived Hypotension Prediction Index to Predict Future Hypotensive Events in Surgical Patients. Anesth. Analg..

[36] Murabito P., Astuto M., Sanfilippo F., La Via L., Vasile F., Basile F., Cappellani A., Longhitano L., Distefano A., Li Volti G. (2022). Proactive Management of Intraoperative Hypotension Reduces Biomarkers of Organ Injury and Oxidative Stress during Elective Non-Cardiac Surgery: A Pilot Randomized Controlled Trial. J. Clin. Med..

[37] Wijnberge M., Geerts B.F., Hol L., Lemmers N., Mulder M.P., Berge P., Schenk J., Terwindt L.E., Hollmann M.W., Vlaar A.P. (2020). Effect of a Machine Learning-Derived Early Warning System for Intraoperative Hypotension vs Standard Care on Depth and Duration of Intraoperative Hypotension During Elective Noncardiac Surgery: The HYPE Randomized Clinical Trial. JAMA.

[38] Chen Y., Guo X., Fu J., Dong T., Liu X., Lv H. (2021). Accuracy of stroke volume variation and pulse pressure variation to predict fluid responsiveness in patients with thoracic kyphosis. Ann. Palliat. Med..

[39] Kim D.H., Shin S., Kim J.Y., Kim S.H., Jo M., Choi Y.S. (2018). Pulse pressure variation and pleth variability index as predictors of fluid responsiveness in patients undergoing spinal surgery in the prone position. Ther. Clin. Risk Manag..

[40] Zlicar M., Novak-Jankovic V., Blagus R., Cecconi M. (2018). Predictive values of pulse pressure variation and stroke volume variation for fluid responsiveness in patients with pneumoperitoneum. J. Clin. Monit. Comput..

[41] Mukai A., Suehiro K., Kimura A., Kodama S., Tanaka K., Mori T., Nishikawa K. (2020). Impact of deep breathing on predictability of stroke volume variation in spontaneous breathing patients. Acta Anaesthesiol. Scand..

[42] Marik P.E., Cavallazzi R., Vasu T., Hirani A. (2009). Dynamic changes in arterial waveform derived variables and fluid responsiveness in mechanically ventilated patients: A systematic review of the literature. Crit. Care Med..

[43] Cannesson M., Vallet B., Michard F. (2009). Pulse pressure variation and stroke volume variation: From flying blind to flying right?. BJA Br. J. Anaesthesia..

[44] Sánchez J.I.A., Ruiz J.D.C., Fernández J.J.D., Ospina-Tascón G.A., Martínez L.E.C. (2020). Use of pulse pressure variation as predictor of fluid responsiveness in patients ventilated with low tidal volume: A systematic review and meta-analysis. Clin. Med. Insights Circ. Respir. Pulm. Med..

[45] De Backer D., Heenen S., Piagnerelli M., Koch M., Vincent J.-L. (2005). Pulse pressure variations to predict fluid responsiveness: Influence of tidal volume. Intensive Care Med..

[46] Doctor M., Siadecki S.D., Cooper D., Rose G., Drake A.B., Ku M., Suprun M., Saul T. (2017). Reliability, Laterality and the Effect of Respiration on the Measured Corrected Flow Time of the Carotid Arteries. J. Emerg. Med..

[47] Abbasi A., Azab N., Nayeemuddin M., Schick A., Lopardo T., Phillips G.S., Merchant R.C., Levy M.M., Blaivas M., Corl K.A. (2020). Change in Carotid Blood Flow and Carotid Corrected Flow Time Assessed by Novice Sonologists Fails to Determine Fluid Responsiveness in Spontaneously Breathing Intensive Care Unit Patients. Ultrasound Med. Biol..

[48] Abbasi A., Nayeemuddin M., Azab N., Schick A., Lopardo T., Phillips G.S., Merchant R.C., Levy M.M., Blaivas M., Corl K.A. (2021). Respiratory Variation in Carotid Artery Peak Systolic Velocity Is Unable to Predict Fluid Responsiveness in Spontaneously Breathing Critically Ill Patients When Assessed by Novice Physician Sonologists. J. Intensive Care Med..

[49] Dai S., Li X., Yang Y., Cao Y., Wang E., Dong Z. (2020). A retrospective cohort analysis for the risk factors of intraoperative hypotension. Int. J. Clin. Pract..

[50] Vaidya G.N., Kolodziej A., Stoner B., Galaviz J.V., Cao X., Heier K., Thompson M., Birks E., Campbell K. (2023). Bedside ultrasound of the internal jugular vein to assess fluid status and right ventricular function: The POCUS-JVD study. Am. J. Emerg. Med..

