# Supplementary material for: Diagnostic Efficacy of Carotid Ultrasound for Predicting the Risk of Perioperative Hypotension or Fluid Responsiveness: A Meta-Analysis
Source: Diagnostics (Basel). 2023 Jul 6;13(13):2290. doi: 10.3390/diagnostics13132290 (PMC10341258; doi:10.3390/diagnostics13132290)

Supplemental Table S1. Search strategies for Medline

1	("General anesthesia" or "Surgery" or "Surgical procedures" or "Regional anesthesia" or "Spinal anesthesia" or "Epidural anesthesia").mp.
2	exp "Anesthesia, General"/ or exp "Anesthesia, Conduction"/ or exp "Surgical Procedures, Operative"/
3	("Carotid artery" or "carotid artery-corrected flow time" or "respiratory variations of peak blood flow velocity" or "respirophasic variation in blood flow peak velocity").mp.
4	exp "Carotid Artery, Common"/
5	("Sonography" or "Echography" or "Ultrasonographic" or "Ultrasonography" or "Ultrasound").mp.
6	exp "Ultrasonography"/
7	("Hypotension" or "Fluid responsiveness" or "Fluid challenge" or "Dehydration" or "hypotensive").mp.
8	(1 or 2) and (3 or 4) and (5 or 6) and 7

Supplemental Figure S1. Deek's funnel plot asymmetry test indicated a low risk of publication bias for the association of corrected blood flow time (FTc) with perioperative hypotension.

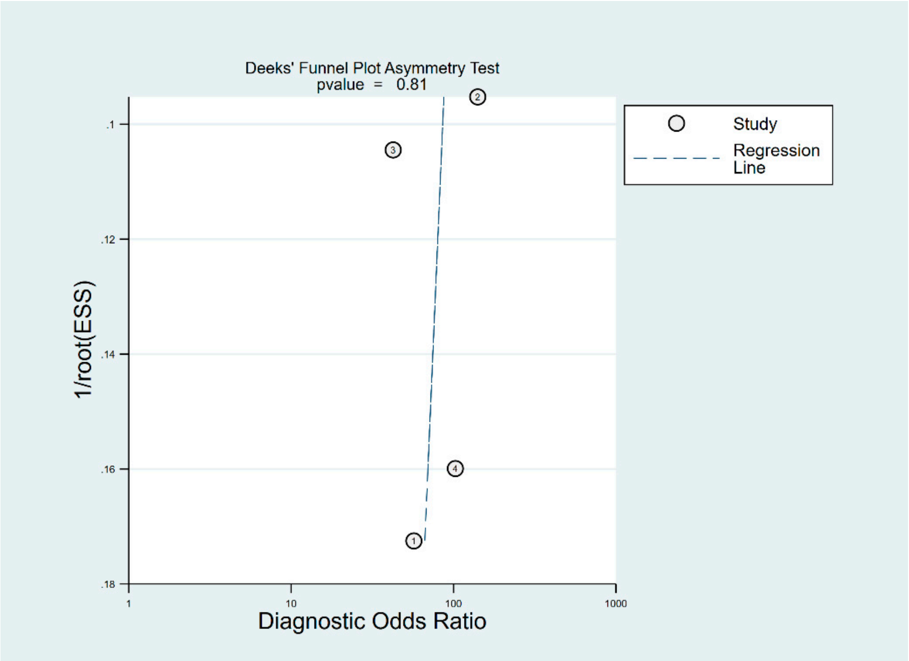

Supplemental Figure S2. Deek's funnel plot asymmetry test indicated a low risk of publication bias for the association of corrected blood flow time (FTc) with perioperative fluid responsiveness.

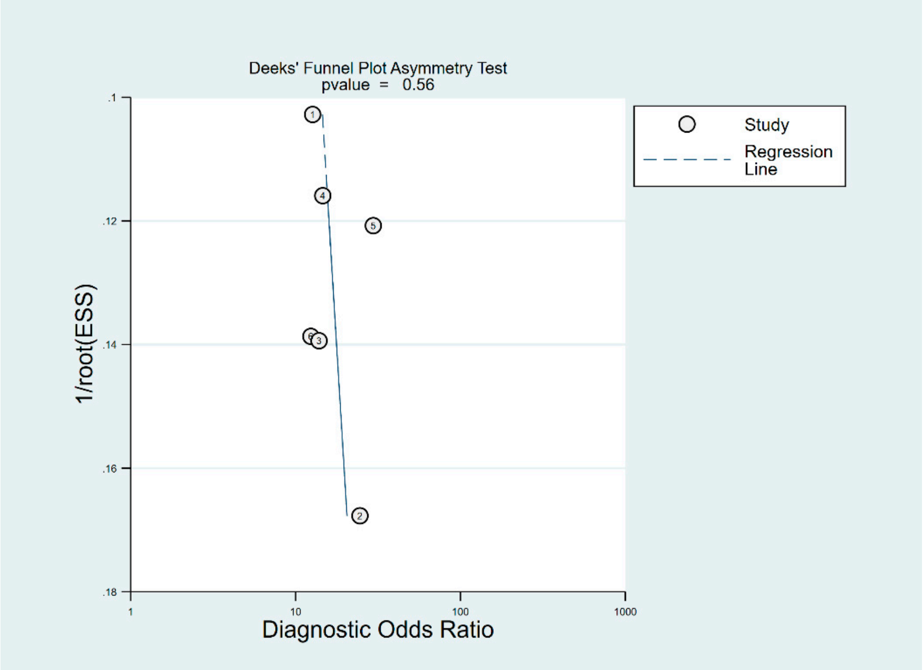

Supplemental Figure S3. Deek's funnel plot asymmetry test indicated a potential risk of publication bias for the association of respirophasic variation in carotid artery blood flow peak velocity (ΔVpeak) with perioperative fluid responsiveness

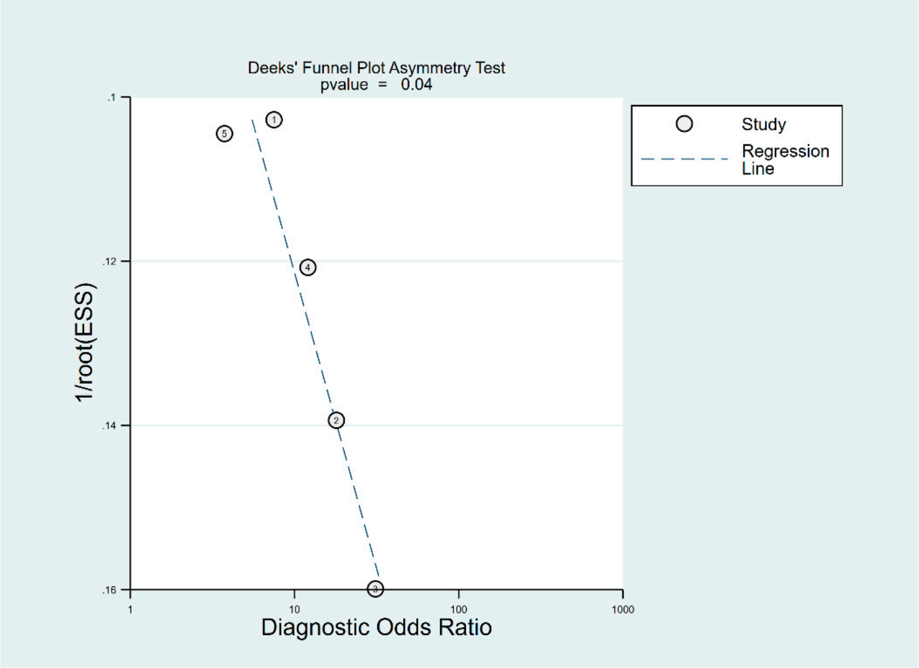

Supplement: Supplementary file 1 [file diagnostics-13-02290-s001.zip › diagnostics-2446233-supplementary.pdf]
